# 

**Published:** 1889-02-16

**Authors:** 


### The Student's Column.

**Suggestions for a Plan of Taking Notes of Medical Cases.***

It is always difficult to draw up a scheme for note-taking that shall be at the same time handy and sufficiently complete. Moreover, a good report is very much a matter of common sense and care. Blessed with these requisites, even the tyro can make a fair report of many cases starting from the question, "How long have you been ill?" or, "What is the matter with you?" and then pursuing one by one the several lines that open out. Nevertheless, he will make fewest mistakes who conducts his examination in a methodical manner, and who, even in what may seem a trivial or obvious case, goes, as a matter of routine, over the whole body to make sure that no latent disease is missed. For this purpose, portable "Suggestions" such as those now before us are valuable, and after a careful perusal of the various headings and details given by Dr. Duffey, they seem to be as complete, and in all respects serviceable as could be. They do not differ much from others that have appeared before, but they are good handy hints, and as such, will certainly prove useful to the studeut. Dr. Duffey's "Suggestions" are printed on stiff paper, and are so conceived and arranged as to be by far the handiest and most useful for students we have ever seen.

*Second Edition. By George F. Duffey, M.D. Dublin: Fannin and Co.

**Notice to Advertisers and Subscribers.**

All Advertisements, Orders for Copies of The Hospital, and Business Communications should be addressed to The Publisher, *not to the Editor*, The Hospital, 38, Old Jewry, E.C.

**To Residents, Secretaries, and Matrons.**

The Editor will be glad to have the Regulations and each Report as issued by Asylums, Hospitals, Convalescent and Nursing Institutions, as well as those of other Charities, and an early copy in duplicate of all Appeals and Festival Notices, etc., addressed to *The Editor, The Lodge, Porchester Square, London, W.* Any superintendent, secretary, matron, or other chief official who regularly sends such information may be registered, on application to the Publisher, to receive free of cost a cover for binding The Hospital in half-yearly volumes.

A Scale of Charges will be found on the first page of the Nursing Supplement.

### Scraps and Gleanings.

New York now boasts a St. Bartholomew's Hospital.

Hunstanton Convalescent Home received 587 patients last year.

Reading Medical Dispensary reports a quiet year of steady work.

Mr. Agnew has promised £200, and Mr. Schwann, M.P., £100, towards

A girl named Madden died of hydrophobia at Middlesex Hospital last week.

The Clifton Dispensary on entering on its 77th year issues a good report.

The Earl of Dartmouth has given a site at Morley for an infectious hospital.

The Manchester Eye Hospital has entered on the seventy-fourth year of its work.

At Stroud General Hospital 265 in-patients and 2,759 out-patients were treated last year.

The Speaker of the House of Commons is vice-president of Warwick Cottage Hospital.

French doctors unite in condemning as unhealthy, the stoves so universally used in Paris.

The Queen has sent a further subscription of £21 to the East London Hospital for Children.

On Thursday last a ball was held at Coventry in aid of St. John's Ambulance Association.

The first cremation in Paris took place last week. Dr. Jacoby had the body of his son cremated.

A Matron writes to the *Standard* to complain of broken, useless toys being sent to the hospitals.

The Flintshire Dispensary treated 1,517 patients last year, of whom 1,236 were cured and 27 died.

A Provident dispensary was opened at Sutton Coldfield, on February 2nd, by the Mayor of the town.

The accounts of Kendall Dispensary show that the medical officers are paid at the rate of 11¾d. a visit.

At the suggestion of Dr. Clements, two duly-qualified midwives are to be appointed to Cork Dispensary.

a Convalescent Fund in connexion with the Pendlebury General Hospital and Dispensary for Sick Children.

Donations are requested for Ashton District Infirmary to meet the expenses of the late enlargements.

At the Royal Ear Hospital, Frith Street, Soho Square, 618 patients were under treatment during January.

The Earl of Derby lately presided at the annual meeting of the governors of the Cotton Districts Convalescent Fund.

Mrs. Scharlieb, M.D., has been appointed one of the three lecturers to the Queen's Jubilee Nursing Association.

The centennial festival of the City Dispensary, London, was held on Wednesday last, the Lord Mayor presiding.

Stockport Infirmary ball took place lately, under the patronage of the Mayor, Lord Egerton, Lord Vernon, and others.

The Dumfries and Galloway Infirmary treated over 400 patients last year; workmen contributed £102 to the expenses.

The Empress Frederick of Germany has expressed her desire to become a vice-president of the British Nurses' Association.

Tavistock Cottage Hospital has issued a satisfactory report. It has treated 71 in-patients and 362 out-patients in the year.

The lease of Bromsgrove Cottage Hospital expires next year, when an attempt will be made to secure more suitable premises.

At Hartlepool Hospital just now the average cost per patient per day is 3s. 7½d., or 3d. less than the average cost quoted last year.

The rules of the Southend Victoria Hospital have been so altered that the General Committee can now appoint or discharge the matron.

On February 2nd the Earl of Hopetoun opened a fever hospital at Drumshorland. It cost £1,550, and will accommodate 12 patients.

Newport Infirmary prides itself on its children's ward, but there is room for other improvements yet. An appeal is made for a Samaritan Fund.

After an exciting discussion, the Chelmsford Sanitary Committee have abandoned the proposed site on Mr. Beach's ground for an infectious hospital.

The Cottage Hospital at Sidmouth owes much of its prosperity to the good offices of the ladies of the town, who take a great interest in its work.

Blackheath and Charlton Cottage Hospital is now complete. The stoves are going to get the building dry, and it will shortly be fit for occupation.

Annual meetings have been numerous at Bath lately, and that excellent institution, the Royal United Hospital, was able to announce a balance in hand.

The medical school in connexion with Victoria University are very anxious for funds to be forthcoming for the completion of Liverpool Royal Infirmary.

At Durham County Hospital last year 445 in-patients and 826 out-patients were treated. Dr. Vann has been appointed consulting physician to the hospital.

The question of lady doctors in Russia has been definitely settled in the affirmative, with the stipulation that they only attend their own sex and young children.

Our readers in Derbyshire are reminded that their presence at the bazaar in aid of the Convalescent Home is required at the Drill Hall, Derby, in the middle of February.

The Empress Frederick and her daughters went all over the hospital ship *Queen Victoria* while she was at Osborne, and specially admired the dispensary and magnificent surgical chest.

The annual report of Kendall Memorial Hospital says that last year was marked by no exceptional circumstances, but by continuance of steady work. Happy is the hospital which has no history

### Amusements and Relaxation.

### NEW PUZZLE PRIZE SCHEME.

SECOND QUARTERLY COMPETITION COMMENCED JANUARY 5th, ENDS MARCH 30th, 1889.

1. Prizes will be awarded for the best Original Puzzles in Prose or Verse relating to any of the Social, Political, or Philanthropical topics of the day. Competitors are requested to *strictly* observe this rule, or their contributions will be discredited.
2. Puzzles may take any form which the ingenuity of the competitors suggest. Answers must accompany contributions, and the source of obscure or ambiguous terms be given.
3. No competitor will be allowed to take more than one prize during the Quarter.
4. Eight Prizes of Standard Books will be given, the choice of the book to be left to the winner. First, Second, Third, Fourth, and Fifth Prizes for best Original Puzzles, £1 1s., 15s., 10s. 6d., 7s. 6d., and 5s. each.
5. First, Second, and Third Prize for Solutions of 15s., 10s. 6d., and 5s. each.

N.B.—All contributions must reach the office, 38, Old Jewry, E.C., not later than the *First Post* on *Thursday* in each week.

### PUZZLES FOR SOLUTION.

**Seventh Week.**

No. 14. Triple Acrostic.

My *first* and my *third* in a great cause unite
To put down my *second*, we all wish they might.

1. My *first* is trying *this* with help from my last,
And so make my *second* a thing of the past.
2. A *short story* of fiction often not good,
And might be improved for pure maidenhood.
3. To be *this* is every man's ambition,
Attained in a moral sense, worthy fruition.
4. The *classical* name of an ancient race,
Near the Sequana was their dwelling-place.
5. Conquered and reconquered, alone this is fame,
France claims me now, and gave me this name.
6. In county Donegal this place you will find,
And thus bring "Ould Ireland" clear to your mind.
7. Now an *adverb* my last, no other would do,
Only this could I find, quite *uninteresting* too.

Gretta.

No. 15. Original Anagram.

Help! Fib not in school of hot persecution.

Crocodile.

No. 16. Square Word.

My *first*, alas, is here once more,
Telling that winter is not o'er.
To sunny *second* would we fly,
'Tis found beneath Italia's sky.
And now my *third* light would you know?
A portent grave of joy or woe.
Here is my *fourth* light with voice loud and strong,
Rending the air as he hurries along.

Nabob.

**Marks for Original Puzzles.**

| Names. | No. of Puzzles sent in Feb. 7. | No. of Marks Feb. 7. | Totals. | Names. | No. of Puzzles sent in Feb. 7. | No. of Marks Feb 7. | Totals. |
|---|---|---|---|---|---|---|---|
| Cotonopolis... | 3 | 6 | 25 | Prior ........... | 3 | 0 | 9 |
| Tinie ............ | 3 | 4 | 25 | Lightowlers . | 2 | 2 | 11 |
| Jenny Wren . | — | — | 4 | Twentieth ... | — | — | 3 |
| Patience ...... | 3 | 6 | 27 | Nabob ......... | 1 | 2 | 2 |
| Padre ......... | — | — | 3 | Crocodile...... | 1 | 3 | 3 |

**Answers.**

No. 10. Arithmorem.

| | | |
|---|---|---|
| B | oomeran | G |
| A | dagi | O |
| L | aryngiti | S |
| F | ran | C |
| O | vermatc | H |
| U | ncl | E |
| R | evisio | N |

No. 11. Original Anagram.

"The Society for the Prevention of Cruelty to Children."

**Marks for Solution of Puzzles.**

Total Marks.—Flora (2), 5; Mater, (2), 3; Scrooge (2), 5; Patience (2), 2; Lady Clara (2), 5; Ruffles (2), 4; Lauriston, 0; Reldas (1), 1; Jenny Wren, 2; Condorcet (1), 1; Tinie (2), 2.

[*The Figures in parentheses are Marks for the week ending Feb. 7th.*]

**Conditions.**

I. Every paper sent in must be clearly written, and one side only must be used. All competitors must mark at the head of their papers the number of their competition.

II. Each paper must be signed by the author with his or her real name and address. A *nom de plume* may be added if the writer does not desire to be referred to by us by his real name. In the case of all prizewinners, however, the real name and address will be published.

III. All communications relating to prize competitions should be addressed to "The Prize Editor, The Hospital, 38, Old Jewry, London, E.C." Competitors are requested not to include in letters, etc., to the Prize Editor, communications on matters of other business. All letters must be fully prepaid.

IV. The Prize Editor of The Hospital is the sole judge of the competitions, and his decision is in all cases final and without appeal.

V. The Prize Editor reserves the right to publish any paper sent in, whether it receives a prize or not. He does not hold himself responsible for any MSS. sent, nor can he undertake to return rejected competitions.

